# Metabolomic estimation of the diagnosis of hepatocellular carcinoma based on ultrahigh performance liquid chromatography coupled with time-of-flight mass spectrometry†

**DOI:** 10.1039/c7ra13616a

**Published:** 2018-03-06

**Authors:** Yuan-Feng Li, Shi Qiu, Li-Juan Gao, Ai-Hua Zhang

**Affiliations:** First Affiliated Hospital, School of Pharmacy, Heilongjiang University of Chinese Medicine Heping Road 24, Xiangfang District Harbin 150040 China yuanfengli7900@126.com aihuatcm@163.com +86-451-86053141 +86-451-86053141

## Abstract

Metabolomics has been shown to be an effective tool for biomarker screening and pathway characterization and disease diagnosis. Metabolic characteristics of hepatocellular carcinoma (HCC) may enable the discovery of novel biomarkers for its diagnosis. In this work, metabolomics was used to investigate metabolic alterations of HCC patients. Plasma samples from HCC patients and age-matched healthy controls were investigated using high resolution ultrahigh performance liquid chromatography-mass spectrometry and metabolic differences were analyzed using pattern recognition methods. 23 distinguishable metabolites were identified. The altered metabolic pathways were associated with arginine and proline metabolism, glycine, serine and threonine metabolism, steroid hormone biosynthesis, starch and sucrose metabolism, *etc*. To demonstrate the utility of plasma biomarkers for the diagnosis of HCC, five metabolites comprising deoxycholic acid 3-glucuronide, 6-hydroxymelatonin glucuronide, 4-methoxycinnamic acid, 11b-hydroxyprogesterone and 4-hydroxyretinoic acid were selected as candidate biomarkers. These metabolites that contributed to the combined model could significantly increase the diagnostic performance of HCC. It has proved to be a powerful tool in the discovery of new biomarkers for disease detection and suggest that panels of metabolites may be valuable to translate our findings to clinically useful diagnostic tests.

## Abstract 2

Metabolomics has been shown to be an effective tool for biomarker screening and pathway characterization and disease diagnosis.

## Graphical abstract

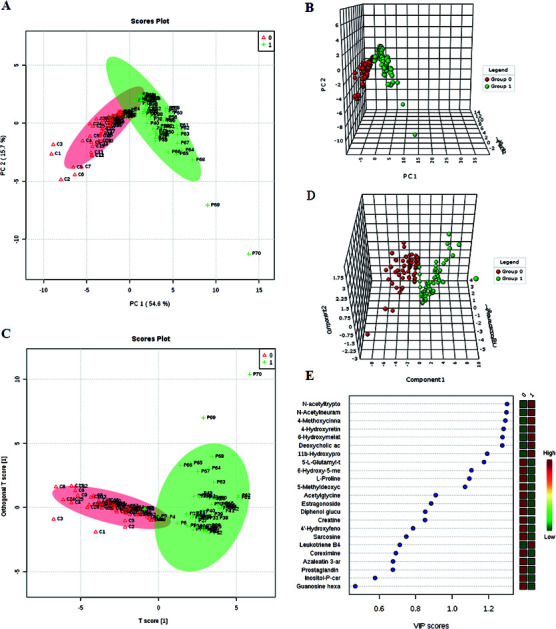

## Introduction

Hepatocellular carcinoma (HCC), is one of the most common malignant tumors in the world and has caused a huge economic and social burden.^1^ Few reliable biomarkers are available in clinical diagnosis to date. The early screening of HCC is an effective strategy to decrease its high mortality. Tissue-based histopathological or blood-based biochemical assays (α-fetoprotein, AFP) in the blood are the major screening methods for HCC.^2^ Recent studies show that AFP lacks sufficient sensitivity,^3^ the quality of the tissue-based histopathological images can be both equipment- and user-dependent.^4^ Further, accurate and early diagnosis of HCC remains a great challenge to date. Therefore, novel screening strategy is still urgently needed for the early discrimination of patients. Recently, metabolomics have been proved to be a highly successful method which could detect metabolic changes in the different pathophysiological status.^5^

Biomarkers can improve disease diagnosis and treatment. There is a close relationship between metabolism and cancer and metabolomics is a promising avenue for biomarker discovery.^6^ Recent reports suggest that metabolite biomarkers have been identified and provided by metabolomic studies.^7^ Metabolomics is an approach that allows the analysis of the entire metabolites in a biological system, and could be used to identify biomarkers for specific pathological status.^8–11^ It has been expanding in scope, from its primary use in clinic for a new diagnostic role in diseases.^12^ Some studies have been reported in biomarker discovery on HCC diseases. The alterations in the normal metabolome may be indicative of diseases.^13–16^ The potential metabolite (biomarker) identification is very important due to it provides diagnostic biomarkers and can help to develop new therapy.

HCC diagnosis is a challenging task for the clinician. In this study, we consider that metabolomics has shown great potential for discovering biomarkers and exploring the metabolic mechanisms of diseases. We aimed to investigating the combined use of metabolites for detection of HCC patients. Metabolites were measured after analysis of plasma samples with ultrahigh performance liquid chromatography coupled to mass spectrometry (UPLC-MS). Multivariate parametric statistical test and receiver operating characteristics analysis were performed to evaluate diagnostic performance of the metabolites. Besides, we will elucidate major pathways contributing to a deeper understanding of the pathological mechanism in HCC.

## Experimental

### Chemicals and reagents

Methanol (HPLC grade) and Acetonitrile (HPLC grade) were purchased from Merck (Darmstodt, Germany). Ultrapure water was provided by a Milli-Q water purification system (Millipore, Billerica, USA). Formic acid and leucine enkephalin was purchased from Sigma-Aldrich (St. Louis, MO, USA).

### Study subjects

The patients with HCC and healthy control subjects were recruited from the First Affiliated Hospital, Heilongjiang University of Chinese Medicine from April 2015 to May 2016, and all patients signed informed consents before the study began. HCC patients were diagnosed according to the histological evidence and the AFP levels were significantly higher in HCC than in healthy control subjects (the AFP level > 400 ng mL^−1^). The detailed information on the clinical information such as age, gender, and some important biochemical indexes for HCC patients and control subjects was presented in ESI Table 1.† A total of 70 patients together with 65 normal control cases were recruited. The study was approved by the Review Board of Heilongjiang University of Chinese Medicine (HUCM-2016-0324) and complied with the provisions of the Good Clinical Practice Guidelines and the Declaration of Helsinki.

### Sample collection and preparation

The whole blood samples were collected in the morning before breakfast from all HCC patients and control group. During this procedure, the plasma was separated by centrifugation at 5000 rpm for 10 min and then immediately stored at −80 °C until further analyses. To ensure the stability and repeatability of UPLC/MS, the blank samples and quality control samples were used in this study. All the plasma samples were thawed at 4 °C and a volume 400 μL of cold methanol were added to 100 μL of plasma for deproteinization, and then centrifuged at 4000 rpm for 10 min. Next, the supernatants were recovered, evaporated using a vacuum rotary dryer and re-suspended in 100 μL acetonitrile/water (1 : 3, v/v), vortex-mixed for 10 min, then centrifuged at 4000 rpm for 10 min, and the supernatant was held for UPLC/MS analysis. To assess instrument stability and quality control sample was used to ensure data quality during the sample sequence. The pooled samples prepared by mixing aliquots of all plasma samples, were injected once after every 10 sample injections *via* the data acquisition process.

### LC-MS analysis

Chromatographic separation was performed on an Acquity UPLC™ system (Waters Corporation, Manchester, UK). Samples were performed on UPLC™ BEH C18 column (50 mm × 2.1 mm i.d., 1.7 μm, Waters, Milford, USA). The column was maintained at 45 °C. The flow rate throughout the separation was maintained at 0.5 mL min^−1^. The mobile phase was consisted of acetonitrile/water (50 : 50 v/v) containing 0.1% formic acid (solvent A) and acetonitrile/water (90 : 10 v/v) containing 0.1% formic acid (solvent B). The gradient conditions were 0–3 min 1–15% B; 3–5 min 15–50% B; 5–9 min 50–95% B; 9–12 min 95% B followed by re-equilibration to the initial starting condition. The samples were kept at 4 °C, and a constant volume of 4 μL was injected for analysis.

MS full scan was performed positive ion mode and negative ion mode using time of flight mass spectrometer (Waters, Milford, USA), which was coupled to the UPLC system. The mass scanning range was 50–1500 *m*/*z* in the full scan mode.

The optimal conditions of MS analysis were as follow: in ESI^+^ mode, the source temperature of 120 °C, the capillary voltage was 3.0 kV, the sampling cone voltage was 30 V, desolvation temperature was 400 °C, desolvation gas flow was 600 L h^−1^; in ESI^−^ mode, the source temperature of 110 °C, the capillary voltage was 2.5 kV, the sampling cone voltage was 20 V, desolvation temperature was 350 °C, desolvation gas flow was 550 L h^−1^. Nitrogen was used as the collision gas at a collision cell pressure of 2.0 × 10^−5^ torr. The flow rates of cone and desolvation gas were set at 60 L h^−1^ and 400 L h^−1^, respectively.

### Data preprocessing and pattern recognition analyses

LC-MS data were analyzed to identify potential discriminant biomarkers. Smoothing, denoising, peak filtering, and alignment of the acquired data were conducted by the MarkerLynx 4.1 application manager (Waters, Manchester, UK). The processed data were imported into MetaboAnalyst 3.0 (http://www.metaboanalyst.ca) for multivariate pattern recognition analysis. PCA was applied to classify plasma samples and performed to detect outliers and distributions of deferent groups and OPLS-DA was carried out to obtain an overview of the complete data set after mean centering scaling. VIP-plot from OPLS-DA was used to rank the differential metabolites and selected for their metabolic pathway analysis, according to their importance to the classification. The differential metabolites were identified based on the MS/MS fragment comparison with the standard compounds, or *via* search of the candidate compounds in the databases including the HMDB, and METLIN.

### Metabolic pathway and network analysis

Pathway analysis was employed to perform the pathway topology analysis on the MetaboAnalyst (http://www.metaboanalyst.ca) to identify the most relevant pathways involved in the HCC. To visualize the metabolic pathway, the correlation network analysis using IPA software was performed.

### Discrimination performance of potential biomarkers

Metabolomics was performed to explore whether metabolomic signatures had the potential discrimination ability between HCC group and control group. To obtain a final diagnostic score, receiver operating characteristic (ROC) curves were generated using the rocplot function program. It allows characterization of diagnostic accuracy, was explored to evaluate the predictive accuracy. ROC curves were used to evaluate the accuracy of the combined signatures model.

### Statistical analysis

Metabolic pathway analysis was employed to perform the pathway topology analysis using the online software MetaboAnalyst 3.0 (http://www.metaboanalyst.ca). The visualization models include PCA and PLS-DA. The areas under curve (AUC) of receiver operating characteristic (ROC) curves were used to determine the diagnostic effectiveness of important metabolites using MetaboAnalyst 3.0. Two-tailed Welch's *t*-tests of covariance were performed using SPSS software (version 19.0; SPSS, Inc., Chicago, IL), with *p* < 0.05 deemed to be significant.

## Results

### Baseline clinical characteristics

Characteristics of the study population are shown in ESI Table 1.† The mean ages, sex, AFP value, HBsAg, ALP, ALT, AST, D-BIL, and T-BIL were not significantly different between patients with HCC cases and age-matched healthy controls. The AFP level indicates a fairly advanced stage of this disease.

### Overall metabolomics analysis

**Fig. 1:**
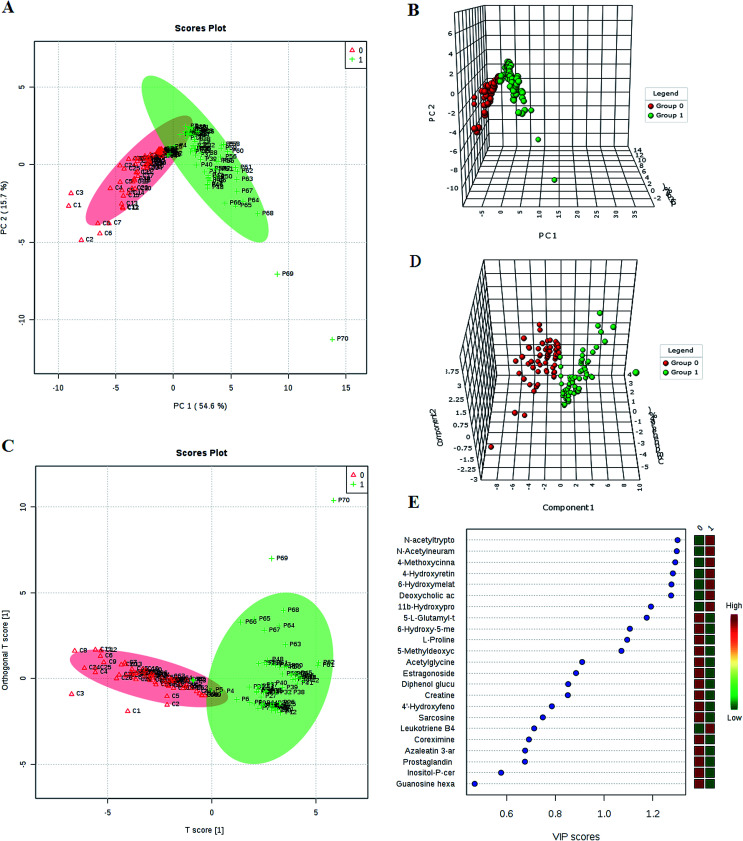
Multivariate analyses screening potential biomarkers. Principal component analysis score plots of the UPLC-MS data from patients (group 1) and controls (group 0) (A) and 3-D model (B). OPLS-DA of the UPLC-MS data from patients (group 1) and controls (group 0) (C) and 3-D model (D). VIP-plot of OPLS-DA for screening of differential metabolites (E).

The first aim was to analyze the metabolome differences between HCC and health cases. To do this, in this study we applied global metabolomics UPLC-MS focusing on the profiles of low-molecular-weight metabolites. Representative base peak intensity chromatograms of plasma samples from the HCC cases and controls were given in Fig. S1.† After alignment and normalization of the data sets of the serum spectra, multivariate statistical analyses were then carried out to enlarge metabolite identification. PCA and 3-D PCA showed clearly distinct distribution in Fig. 1A and B, respectively, which implied abnormal metabolic pattern from HCC patients. Orthogonal projection to latent structure with discriminant analysis (OPLS-DA) was then subsequently used to determine differential metabolites responsible for metabolic differences. The OPLS-DA model after excluding the outliers observed in the PCA reveal an obvious separation between groups (Fig. 1C and D).

### Differential metabolites analysis

**Fig. 2:**
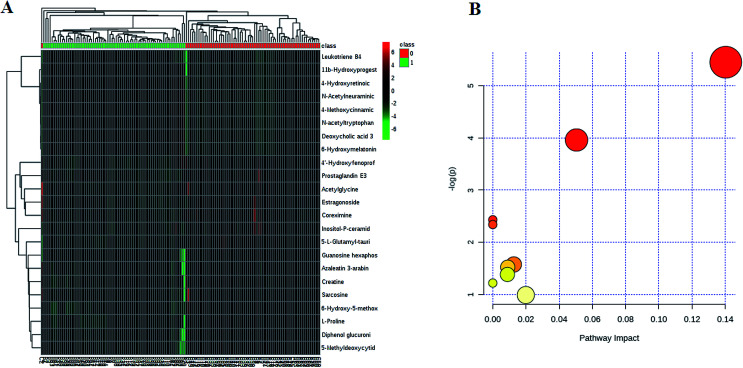
Plasma metabolites and metabolic pathways related to HCC. The heatmap visualization for metabolites statistically significant with a potential identity (A), as analyzed by MetaboAnalyst. Summary of metabolic pathways of significantly changed metabolites with MetPA (B). Circles represent metabolism (ESI Table S3†).

We employed an OPLS-DA statistical approach termed VIP-plot to select metabolites that highly contributed to the behaviors. VIP-plot of controls *vs.* HCC patients was shown in Fig. 1E, which is a scatter plot that combines the covariance and correlation. Higher values of VIP indicate metabolites that are more important to the classification. A *T* test was performed in variables with significant differences between HCC cases and control individuals (*P* < 0.05) were kept. As a result, a total of 23 discriminate variables (listed in ESI Table 2†) as interesting candidates were consistently altered in HCC. Elemental composition was calculated using the Masslynx 4.1 tool. Meanwhile, it was finally confirmed by comparison with a standard sample. Then, these changes of the metabolites are further visualized in the heatmap and indicated that the two groups could be separated based on these metabolites (Fig. 2A).

### Metabolic pathways analysis

**Fig. 3:**
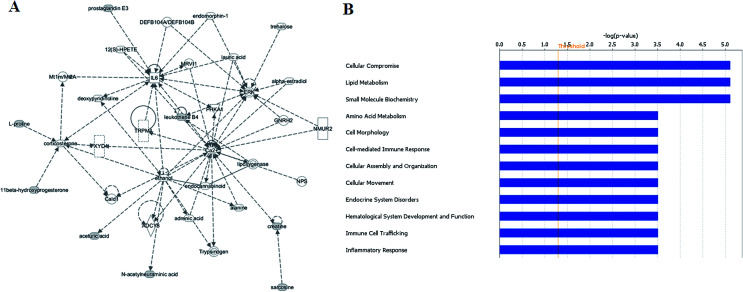
Ingenuity pathway analysis on the significantly changed metabolites. Ingenuity pathway analysis (A) and functional pathway category analysis (B).

All of the differential metabolites were determined by enrichment analysis utilizing MetaboAnalyst 3.0. The biological pathway analysis results showed 4 metabolic pathways, including arginine and proline metabolism, glycine, serine and threonine metabolism, steroid hormone biosynthesis, starch and sucrose metabolism were the most influenced metabolic pathway which set as a pathway impact >0.01 (Fig. 2B and ESI Table 3†). To visualize biological connectivity of the significantly changed metabolites, the network-generating algorithm of ingenuity pathway analysis was used to maximize the interconnectedness of molecules in the HCC-related metabolic network (Fig. 3A). Functional pathway category analysis of differential metabolites related to HCC was carried out. Consequently, cellular compromise, lipid metabolism, small molecule biochemistry pathways were considered closely related to the development of HCC. It revealed that the HCC patients possessed a highly unique metabolic phenotype characterized by these differential metabolites (Fig. 3B and ESI Table 4†).

### Potential diagnostics estimation

**Fig. 4:**
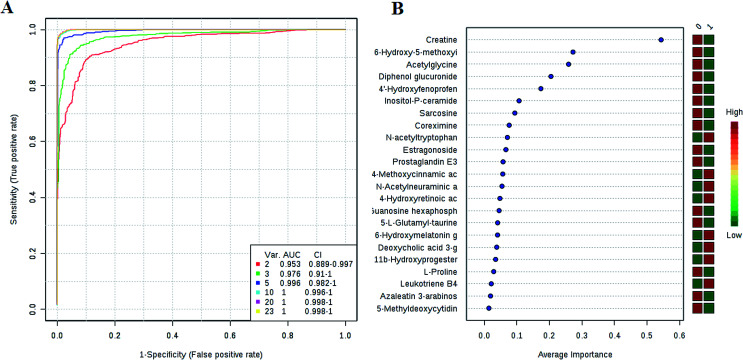
Evaluation of potential biomarkers. A combination metabolites model calculated from the logistic regression analysis (A); features ranked by their contributions to classification accuracy (B).

We have performed ROC analysis to further characterize the predictive value of these individual metabolites independently. We found 7 metabolites including deoxycholic acid 3-glucuronide, 6-hydroxymelatonin glucuronide, 4-methoxycinnamic acid, 11b-hydroxyprogesterone, 4-hydroxyretinoic acid, *N*-acetylneuraminic acid, *N*-acetyltryptophan with an area under the curve (AUC) < 0.94 (ESI Table 5†). To improve the prediction of HCC, a combination of more than one discriminatory metabolite was developed *via* logistic regression analysis (Fig. 4A). We found that the combination of metabolites was a better discriminator (AUC > 95%) than each metabolite individually (AUC < 94%), which reinforced the improved capacity of biomarker patterns to distinguish different groups. Thus, the distinctive signatures with the 5 metabolites had achieved the highest AUC value and significantly increased the diagnostic performance of HCC, achieving an AUC value of 0.996. To validate the importance of these metabolites from the ROC analysis and to further screen out a group of metabolites as potential biomarkers for accurately diagnostics. Notably, for the feature ranking method, top 5 metabolites including deoxycholic acid 3-glucuronide, 6-hydroxymelatonin glucuronide, 4-methoxycinnamic acid, 11b-hydroxyprogesterone, and 4-hydroxyretinoic acid contributed to the combined model (Fig. 4B). Thus, these distinctive signatures greatly improved the diagnostic performance of a single metabolite in HCC.

## Discussion

Many HCC patients are diagnosed at an advanced stage. The early detection of HCC has been a great challenge until now and the developing new effective methods for the discovery of novel biomarkers for HCC are urgently needed. Reliable biomarkers for the diagnosis of HCC need to reduce mortality and therapeutic expenditure. Metabolites can be changed in cancer, because the metabolites produced are indicators of what is happening in the metabolism of disease conditions.^17,18^ Metabolomics has been introduced as a way for finding helpful diagnostic biomarkers for the clinician. Recently, some previous studies have attempted to use metabolomics to discover biomarkers that might apply the detection of cancer.^19^ Identification of dysfunctional metabolic pathways of cancer *via* metabolomics can be used to discover biomarkers.^20^ Numerous studies have reported the dysregulated metabolism is associated with the survival of cancers in patients.^21^ A growing number of studies have used plasma-based metabolomics as a method of discovering biomarkers for diagnosing HCC.^22^

In the present study, a non-targeted UPLC/MS plasma metabolism method was utilized to explore the metabolic characteristics related to the HCC patients and to screen meaningful predictors. A comprehensive workflow was employed to determinate potential biomarkers, including the visualization of samples and metabolites, multivariate screening for the classification of disease status and ROC validation. We compared plasma metabolic profiles of 70 patients with HCC to identify its metabolic signatures. PCA showed clearly different distribution that was caused by different levels of metabolites. We employed VIP-plot of OPLS-DA statistical approach to select metabolites that highly contributed to the sharp separation behaviors. A total of 23 metabolites were defined as biomarker candidates. We identified 12 metabolites in ESI^+^ mode and 11 metabolites in ESI^−^ mode related to the HCC. In this study, we provided evidence that there is a high relationship between these metabolites and HCC. Pathway analysis has revealed that arginine and proline metabolism, glycine, serine and threonine metabolism, steroid hormone biosynthesis, starch and sucrose metabolism and so on were most relevantly disturbed, and gives a better understanding of the potential mechanism of HCC.

In recent years, numerous findings have been published that using the marker candidates could potentially improve the diagnosis of patients.^23–26^ Establishing a diagnostic model to predict the HCC was difficult due to the distinct metabolic profile of HCC consisting of the 4 altered metabolic pathways and 23 corresponding metabolites. Subsequently, we then performed ROC analysis to characterize the predictive value of these metabolites for discriminating HCC. Evaluation of biomarkers by ROC analysis showed that 13 metabolites with high AUC above 0.90. Their AUC values indicated a satisfactory performance in validation data sets. A combination of more than one discriminatory metabolite was developed *via* logistic regression analysis to construct an effective diagnostic model for HCC. Especially, for the feature ranking method, interestingly, we observed that top 5 metabolites including deoxycholic acid 3-glucuronide, 6-hydroxymelatonin glucuronide, 4-methoxycinnamic acid, 11b-hydroxyprogesterone and 4-hydroxyretinoic acid contributed to the combined model and had achieved the highest AUC value. These metabolites significantly increased the diagnostic performance of HCC. The potential biomarker pattern may has the advantages of improving the diagnostic performance, as well as simplifying the practical application. It revealed the potential pathogenesis of HCC, and also provided a feasible diagnostic tool for HCC populations through detection of plasma metabolites.

The development of biomarkers to diagnose HCC is meaningful for both patient care and research. In this study, we had investigated whether the alterations in plasma metabolites are available for detection of HCC, and performed based on the UPLC/MS technique supported by the advanced chemometric analysis. The aim of this study is to find metabolite biomarkers that would allow the discovery and diagnosis of HCC. We found that the combination of metabolites was a better discriminator than each metabolite individually. In this study, the plasma metabonomics analysis for identifying potential biomarkers to diagnose HCC was successfully demonstrated, which has the advantages of reliable, simple, and low-cost. It demonstrates the efficacy and the potential of plasma metabolomics strategy for biomarker discovery, and providing novel insights for disease study. In conclusion, our research highlights the high-throughput metabolomics makes it an ideal methodology for rapidly identifying the global metabolic alterations associated with HCC-alterations that not only enhance our understanding of the metabolic mechanism, but that can improve HCC diagnostics.

## Conclusions

In this study, a non-targeted UPLC-TOFMS metabolomics method in conjunction with pattern recognition analyses based on 65 plasma samples from healthy controls, and 70 plasma samples from HCC patients were established to explore the metabolic characteristics of HCC. We applied plasma metabolomics approach based UPLC-MS combined with pattern recognition approach for identifying potential novel diagnostic biomarkers of HCC. Interestingly, we observed that 23 potential biomarkers in the HCC subjects were quite different from the control subjects. Additionally, significant metabolic pathway alterations were observed in the HCC, including arginine and proline metabolism, glycine, serine and threonine metabolism, steroid hormone biosynthesis, starch and sucrose metabolism. Subsequently, the diagnostic potential of these biomarkers was then carefully evaluated based on the ROC curve with the AUC value. The combinational use of top five metabolites has the promising clinical potential to improve the diagnostic performance of HCC. More studies are still required for further large-scale validation. Overall, however, our study indicates that plasma metabolomics is a powerful resource to explore the disease mechanism and discover the potential biomarkers for diagnosis in clinic.

## Conflicts of interest

The authors declare no competing financial interests.

## Supplementary Material

RA-008-C7RA13616A-s001
