# Supplementary material for: Metabolomic estimation of the diagnosis of hepatocellular carcinoma based on ultrahigh performance liquid chromatography coupled with time-of-flight mass spectrometry
Source: RSC Adv. 2018 Mar 6;8(17):9375–82. doi: 10.1039/c7ra13616a (PMC9078651; doi:10.1039/c7ra13616a)
Supplement: RA-008-C7RA13616A-s001 [file RA-008-C7RA13616A-s001.pdf]

Electronic Supplementary Material (ESI) for RSC Advances.
This journal is © The Royal Society of Chemistry 2018

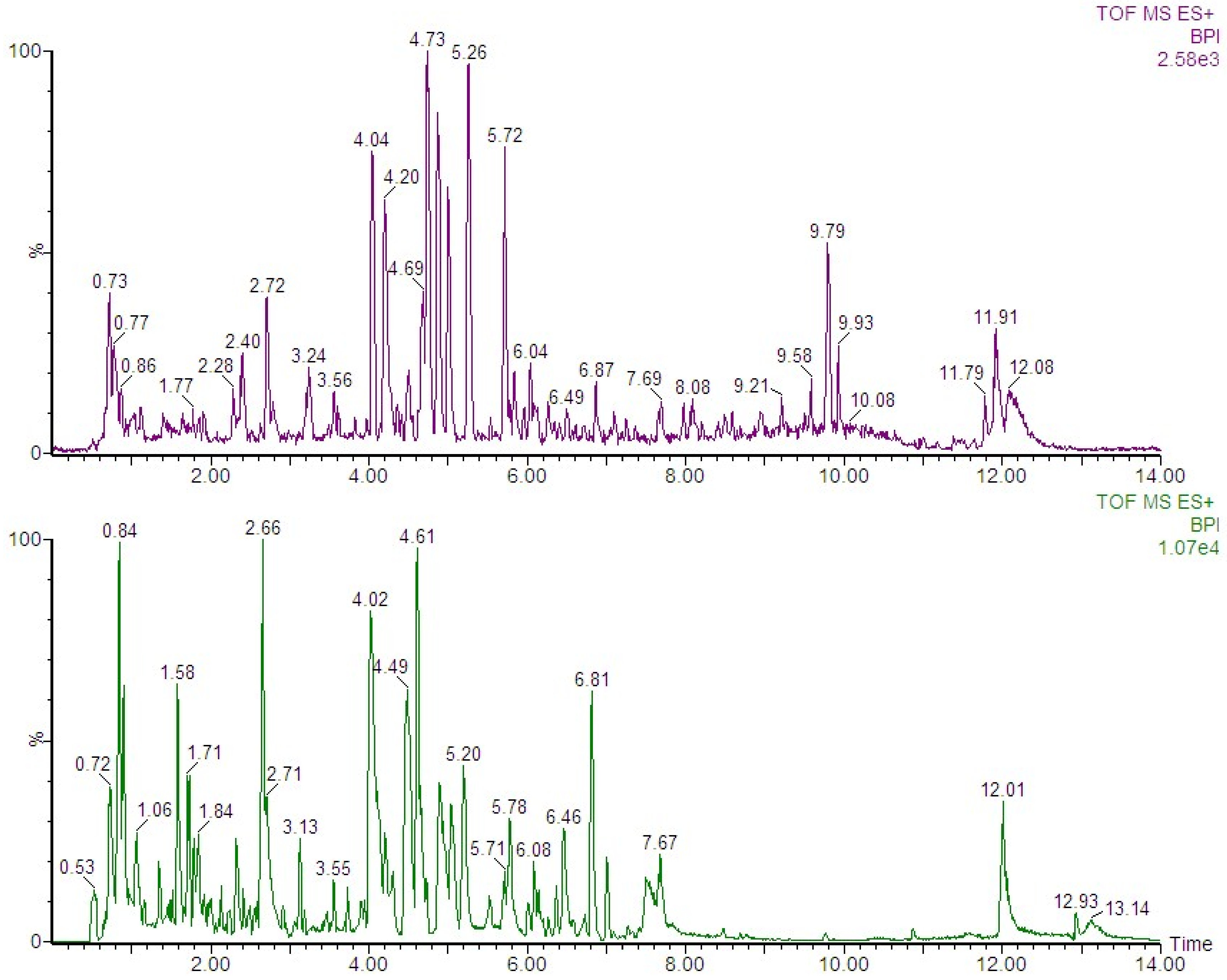

Fig.S1. Typical base peak chromatograms of plasma samples from control subjects (up) and HCC patients (down) obtained by UPLC-MS.

Table S1. Clinical characteristics of healthy humans and HCC patients.

**Parameters**	**Healthy Controls (n = 65)**	**HCC Patients (n =70)**
Age	55.4 ± 8.5	56.3 ± 6.7
Male/Female	36/29	37/33
AFP value (ng/mL)	20 ± 1.3	454.7 ± 187.4
HBsAg (positive %)	-	100
ALP (IU/L)	88.0 ± 16.7	164.9 ± 31.4
ALT (IU/L)	16.2 ± 5.3	75.4 ± 15.1
AST (IU/L)	26.9± 13.7	79.0± 12.6
D-BIL (μmol/L)	2.0 ± 0.8	6.4 ± 1.3
T-BIL (μmol/L)	8.5 ± 2.1	19.6 ± 5.3

Note: AFP, alpha-fetoprotein; HBsAg, hepatitis B surface antigen; ALP, alkaline phosphatase; ALT, alanine aminotransferase; AST, aspartate transaminase; D-BIL, direct bilirubin; T-BIL, total bilirubin. Data are presented as mean ± SE.

Table S2. Information of plasma metabolites related to HCC detected by UPLC/MS.

No	Rt (min)	m/z	Compound ID	Adducts	Formula	Mass Error (ppm)	Description	Anova (p)
1	11.00	1010.9677	HMDB01541	M-H	C20H30N10O26P6	-0.84	Guanosine hexaphosphate adenosine	0.0076
2	0.50	952.6796	HMDB12237	M-H	C50H100NO13P	-0.67	Inositol-P-ceramide	0.0354
3	16.77	567.3154	HMDB02596	M-H	C30H48O10	-0.57	Deoxycholic acid 3-glucuronide	0.0322
4	10.20	477.1046	HMDB33650	M-H	C22H22O12	0.51	Estragonoside	0.0000
5	9.00	447.0942	HMDB37359	M-H	C21H20O11	0.06	Azaleatin 3-arabinoside	0.0000
6	4.86	433.1149	HMDB60772	M-H	C21H22O10	0.94	4'-Hydroxyfenoprofen glucuronide	0.0213
7	10.94	423.1360	HMDB60786	M-H	C19H24N2O9	-0.51	6-Hydroxymelatonin glucuronide	0.0000
8	10.39	355.1205	HMDB02040	2M-H	C10H10O3	0.88	4-Methoxycinnamic acid	0.0000
9	6.70	116.0366	HMDB00532	M-H	C4H7NO3	1.92	Acetylglycine	0.0100
10	0.91	88.0401	HMDB00271	M-H	C3H7NO2	-0.28	Sarcosine	0.0500
11	7.20	653.2793	HMDB30286	2M-H	C19H21NO4	-0.57	Coreximine	0.0100
12	12.86	349.2021	HMDB02664	M+H	C20H32O5	0.09	Prostaglandin E3	0.0000
13	5.87	338.0883	HMDB10362	M+H	C15H19NO8	0.51	6-Hydroxy-5-methoxyindole glucuronide	0.0128
14	16.05	335.2236	HMDB01085	M+H	C20H34O4	0.31	Leukotriene B4	0.0014
15	14.64	331.1923	HMDB04031	M+H	C20H30O4	0.44	11b-Hydroxyprogesterone	0.0100
16	17.83	315.1968	HMDB06254	M+H	C20H30O3	0.79	4-Hydroxyretinoic acid	0.0007
17	4.82	308.0983	HMDB00230	M+H	C11H21NO9	-0.17	N-Acetylneuraminic acid	0.0027
18	0.72	285.0633	HMDB59998	M+H	C12H16O8	0.96	Diphenol glucuronide	0.0053
19	7.64	261.1340	HMDB00064	2M+H	C4H11N3O2	0.88	Creatine	0.0000
20	10.23	253.0505	HMDB04195	M+H	C7H16N2O6S	1.02	5-L-Glutamyl-taurine	0.0074
21	7.67	245.0931	HMDB13713	M+H	C13H16N2O3	-0.45	N-acetyltryptophan	0.0261
22	2.72	229.1189	HMDB00162	2M+H	C5H11NO2	-0.96	L-Proline	0.0069
23	8.36	483.2075	HMDB02224	2M+H	C10H17N3O4	0.68	5-Methyldeoxycytidine	0.0100

Table S3 Summary of metabolic pathways of significantly changed metabolites with MetPA.

**Pathways**	**Total**	**Expected**	**Hits**	**Raw p**	**Holm adjust**	**FDR**	**Impact**
Arginine and proline metabolism	77	0.3519	3	0.0043	0.34535	0.34535	0.1403
Glycine, serine and threonine metabolism	48	0.2194	2	0.0191	1	0.764	0.0504
Taurine and hypotaurine metabolism	20	0.0914	1	0.0879	1	1	0
Retinol metabolism	22	0.1005	1	0.0963	1	1	0
Starch and sucrose metabolism	50	0.2285	1	0.2066	1	1	0.0127
Pentose and glucuronate interconversions	53	0.2422	1	0.2176	1	1	0.0090
Arachidonic acid metabolism	62	0.2833	1	0.2500	1	1	0.0088
Aminoacyl-tRNA biosynthesis	75	0.3428	1	0.2946	1	1	0
Steroid hormone biosynthesis	99	0.4524	1	0.3706	1	1	0.0199

Table S4. Functional pathway category analysis

Category	p-value	Molecules
Cellular Compromise	7.82E-06-3.66E-02	L-proline,leukotriene B4,creatine
Lipid Metabolism	7.82E-06-3.99E-02	L-proline,leukotriene B4,11beta-hydroxyprogesterone,creatine
Small Molecule Biochemistry	7.82E-06-4.67E-02	L-proline,sarcosine,leukotriene B4,11beta-hydroxyprogesterone, creatine
Amino Acid Metabolism	3.1E-04-5.56E-03	sarcosine,creatine
Cell Morphology	3.1E-04-1.26E-02	leukotriene B4,creatine
Cell-mediated Immune Response	3.1E-04-2.33E-02	leukotriene B4
Cellular Assembly and Organization	3.1E-04-3.15E-02	leukotriene B4,creatine
Cellular Movement	3.1E-04-4.88E-02	prostaglandin E3,sarcosine,leukotriene B4,creatine
Endocrine System Disorders	3.1E-04-6.18E-03	prostaglandin E3,leukotriene B4,creatine
Hematological System Development and Function	3.1E-04-4.88E-02	leukotriene B4,creatine,N-acetylneuraminic acid
Immune Cell Trafficking	3.1E-04-4.88E-02	leukotriene B4,creatine
Inflammatory Response	3.1E-04-3.48E-02	leukotriene B4,creatine
Neurological Disease	3.1E-04-4.85E-02	L-proline,leukotriene B4,creatine, N-acetylneuraminic acid
Organ Morphology	3.1E-04-2.12E-02	leukotriene B4,creatine
Organismal Injury and Abnormalities	3.1E-04-4.85E-02	prostaglandin E3,L-proline,leukotriene B4, creatine,N-acetylneuraminic acid
Skeletal and Muscular System Development and Function	3.1E-04-4.26E-02	creatine
Tissue Morphology	3.1E-04-1.93E-02	creatine
Cell Death and Survival	6.19E-04-1.66E-02	L-proline,creatine
Drug Metabolism	6.19E-04-4.08E-02	creatine
Gastrointestinal Disease	6.19E-04-3.39E-02	prostaglandin E3,creatine
Hematopoiesis	6.19E-04-8.64E-03	leukotriene B4,creatine,N-acetylneuraminic acid
Hypersensitivity Response	6.19E-04-2.12E-02	leukotriene B4,creatine
Nervous System Development and Function	6.19E-04-4.26E-02	L-proline,sarcosine,creatine
Skeletal and Muscular Disorders	6.19E-04-4.05E-02	creatine,N-acetylneuraminic acid
Cellular Growth and Proliferation	9.29E-04-1.32E-02	L-proline,prostaglandin E3,sarcosine,leukotriene B4
Inflammatory Disease	9.29E-04-7.41E-03	leukotriene B4,creatine
Organismal Development	9.29E-04-3.09E-02	L-proline,leukotriene B4,creatine
Protein Synthesis	9.29E-04-1.57E-02	L-proline,leukotriene B4,creatine
Respiratory Disease	9.29E-04-3.74E-03	leukotriene B4,creatine
Cell-To-Cell Signaling and Interaction	1.24E-03-4.08E-02	L-proline,leukotriene B4,creatine, N-acetylneuraminic acid
Metabolic Disease	1.24E-03-4.64E-03	leukotriene B4,creatine
Cancer	1.55E-03-2.03E-02	prostaglandin E3,N-acetylneuraminic acid
Cardiovascular System Development and Function	1.55E-03-1.55E-03	creatine
Hair and Skin Development and Function	1.55E-03-3.03E-02	sarcosine,leukotriene B4
Free Radical Scavenging	1.59E-03-4.55E-02	L-proline,leukotriene B4,creatine
Carbohydrate Metabolism	1.86E-03-4.67E-02	leukotriene B4,creatine,N-acetylneuraminic acid
Hereditary Disorder	1.86E-03-4.05E-02	leukotriene B4,creatine,N-acetylneuraminic acid
Molecular Transport	1.86E-03-4.67E-02	L-proline,sarcosine,leukotriene B4,creatine
Nutritional Disease	2.17E-03-2.17E-03	creatine
Cell Cycle	2.47E-03-3.09E-03	L-proline
Hepatic System Development and Function	2.47E-03-3.71E-03	L-proline,leukotriene B4
Hepatic System Disease	2.47E-03-2.47E-03	creatine
Lymphoid Tissue Structure and Development	3.09E-03-3.09E-03	leukotriene B4
Developmental Disorder	3.4E-03-4.05E-02	leukotriene B4,creatine,N-acetylneuraminic acid
Energy Production	3.4E-03-2E-02	creatine
Nucleic Acid Metabolism	3.4E-03-3.24E-02	leukotriene B4,creatine
Psychological Disorders	3.4E-03-1.63E-02	leukotriene B4,creatine
Behavior	3.71E-03-3.71E-03	creatine
Digestive System Development and Function	3.71E-03-3.71E-03	leukotriene B4
Organ Development	3.71E-03-3.71E-03	leukotriene B4
Organismal Functions	3.71E-03-3.78E-02	leukotriene B4,creatine
Reproductive System Development and Function	4.33E-03-4.33E-03	leukotriene B4
Endocrine System Development and Function	4.64E-03-4.64E-03	L-proline
Cellular Development	5.37E-03-1.32E-02	prostaglandin E3,sarcosine,leukotriene B4,creatine
Tumor Morphology	6.18E-03-6.18E-03	prostaglandin E3
Renal and Urological Disease	7.41E-03-8.64E-03	creatine
Cellular Function and Maintenance	8.02E-03-4.11E-02	leukotriene B4,creatine
Cardiovascular Disease	8.64E-03-2.94E-02	leukotriene B4,creatine

Connective Tissue Development and Function	8.64E-03-8.64E-03	creatine
DNA Replication, Recombination, and Repair	8.64E-03-3.15E-02	creatine
Tissue Development	8.64E-03-8.64E-03	creatine
Cell Signaling	1.63E-02-3.24E-02	leukotriene B4,creatine
Vitamin and Mineral Metabolism	1.63E-02-1.93E-02	leukotriene B4,creatine
Embryonic Development	2.97E-02-2.97E-02	sarcosine
Renal and Urological System Development and Function	3.48E-02-3.48E-02	sarcosine

Table S5. ROC curve analysis of potential biomarkers in HCC.

No	Metabolite	AUC	T-tests	Log2 FC
1	Deoxycholic acid 3-glucuronide	0.9362	5.34E-28	-3.9683
2	6-Hydroxymelatonin glucuronide	0.9340	4.27E-28	-4.1299
3	4-Methoxycinnamic acid	0.9327	3.47E-29	-4.1143
4	11b-Hydroxyprogesterone	0.9319	4.62E-23	-4.6511
5	4-Hydroxyretinoic acid	0.9316	1.68E-28	-4.1871
6	N-Acetylneuraminic acid	0.9303	1.28E-29	-4.2756
7	N-acetyltryptophan	0.9301	7.39E-30	-4.2299
8	6-Hydroxy-5-methoxyindole glucuronide	0.9299	6.16E-19	4.3730
9	5-L-Glutamyl-taurine	0.9298	3.68E-22	4.2142
10	5-Methyldeoxycytidine	0.9252	1.69E-17	4.2020
11	L-Proline	0.9223	1.81E-18	4.2110
12	Acetylglycine	0.9138	4.08E-12	4.0813
13	Leukotriene B4	0.9077	1.62E-07	2.0671
14	Diphenol glucuronide	0.8986	1.37E-10	4.2491
15	Creatine	0.8866	1.49E-10	4.2471
16	Sarcosine	0.8615	3.19E-08	4.1774
17	Estragonoside	0.8477	2.00E-11	4.0628
18	4'-Hydroxyfenoprofen glucuronide	0.8154	5.19E-09	4.0231
19	Azaleatin 3-arabinoside	0.8104	7.83E-07	4.0316
20	Coreximine	0.7668	4.09E-07	4.0219
21	Guanosine hexaphosphate adenosine	0.7576	0.000812106	4.0378
22	Prostaglandin E3	0.7556	8.07E-07	3.9910
23	Inositol-P-ceramide	0.7022	3.09E-05	3.9865
